# Development and validation of prediction model for early warning of ovarian metastasis risk of endometrial carcinoma

**DOI:** 10.1097/MD.0000000000035439

**Published:** 2023-10-13

**Authors:** Qin Zhao, Yinuo Li, Tiejun Wang

**Affiliations:** a Key Laboratory of Cancer Invasion and Metastasis, Ministry of Education, Department of Gynecology, National Clinical Research Center for Obstetrical and Gynecological Diseases, Tongji Hospital, Tongji Medical College, Huazhong University of Science and Technology, Wuhan, China; b Department of Breast Surgery, Hubei Cancer Hospital, Tongji Medical College, Huazhong University of Science and Technology, Hubei Provincial Clinical Research Center for Breast Cancer, Wuhan Clinical Research Center for Breast Cancer, Wuhan, China.

**Keywords:** endometrial carcinoma, machine learning, ovarian metastasis, prediction, risk

## Abstract

Ovarian metastasis of endometrial carcinoma (EC) patients not only affects the decision of the surgeon, but also has a fatal impact on the fertility and prognosis of patients. This study aimed build a prediction model of ovarian metastasis of EC based on machine learning algorithm for clinical diagnosis and treatment management guidance. We retrospectively collected 536 EC patients treated in Hubei Cancer Hospital from January 2017 to October 2022 and 487 EC patients from Tongji Hospital (January 2017 to December 2020) as an external validation queue. The random forest model, gradient elevator model, support vector machine model, artificial neural network model (ANNM), and decision tree model were used to build ovarian metastasis prediction model for EC patients. The predictive efficacy of 5 machine learning models was evaluated by receiver operating characteristic curve and decision curve analysis. For screening of candidate predictors of ovarian metastasis of EC, the degree of tumor differentiation, lymph node metastasis, CA125, HE4, Alb, LH can be used as a potential predictor of ovarian metastasis prediction model in EC patients. The effectiveness of the prediction model constructed by the 5 machine learning algorithms was between (area under curve [AUC]: 0.729, 95% confidence interval [CI]: 0.674–0.784) and (AUC: 0.899, 95% CI: 0.844–0.954) in the training set and internal verification set, respectively. Among them, the ANNM was equipped with the best prediction effectiveness (training set: AUC: 0.899, 95% CI: 0.844–0.954) and (internal verification set: AUC: 0.892, 95% CI: 0.837–0.947). The prediction model of ovarian metastasis of EC patients based on machine learning algorithm can achieve satisfactory prediction efficiency, among which ANNM is the best, which can be used to guide clinicians in diagnosis and treatment and improve the prognosis of EC patients.

## 1. Introduction

Endometrial carcinoma (EC), as one of the 3 most common gynecological malignant tumors in the world, is an epithelial malignant tumor that occurs in the endometrium.^[1,2]^ To date, in order to prevent tumor metastasis and recurrence, surgery is the most important treatment method for EC.^[3]^ In clinical practice, it is more commonly used to treat EC by total hysterectomy with ovariectomy and pelvic lymph node dissection. Alarmingly, in recent years, the disease has become younger. About 25% of patients are premenopausal women, and up to 5% of women under 40 years old.^[4,5]^ In view of this, whether ovariectomy has become the focus of surgical controversy, and only the trauma caused by hysterectomy also has a great impact on patients’ reproductive function.

Previous research reports have shown that the probability of ovarian metastasis of EC is 1.7% to 11.0%.^[6,7]^ Given this situation, if we can find out the risk factors of ovarian metastasis of EC, it will help us to take early intervention measures and avoid the trauma caused by ovariectomy or surgery. However, the current research reports on the risk factors of ovarian metastasis of EC are based on the indicators observed during operation, and the views are inconsistent.^[7–9]^ Therefore, building a prediction model for ovarian metastasis of EC through some preoperative indicators will help low-risk patients choose other treatment methods to avoid trauma caused by surgery.

In recent years, machine learning is a common research hotspot in the field of artificial intelligence and pattern recognition.^[10,11]^ It can centrally learn and analyze large-scale and complex data through different computing methods, and has incomparable application value in clinical disease diagnosis and prognosis evaluation.^[11,12]^ Encouraged by this, this study intends to explore the risk factors of ovarian metastasis of EC and build a predictive model of ovarian cancer metastasis based on machine learning, combined with traditional clinical pathological parameters and serological related indicators, and carry out hierarchical classification, refined and individual management for such patients, as well as help improve the prognosis and survival of patients with EC and ovarian metastasis.

## 2. Materials and methods

### 2.1. Study population

**Figure 1.**
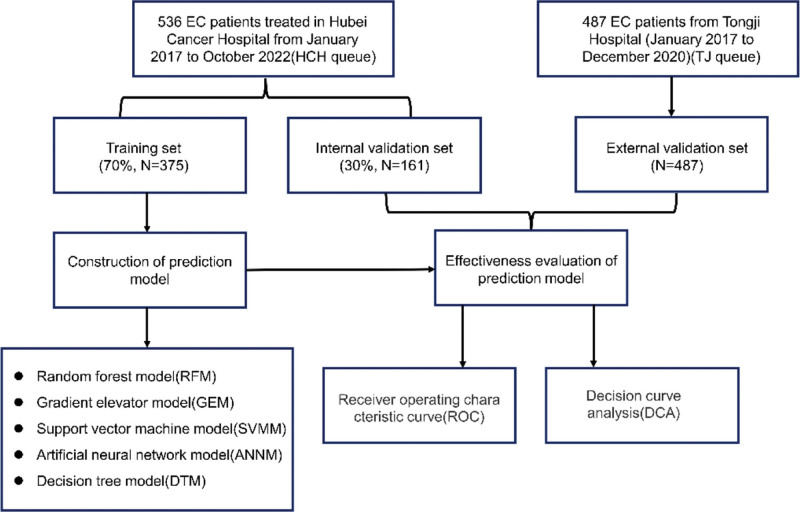
Flow chart of patient inclusion, model construction and verification.

We included 536 EC patients who were treated in Hubei Cancer Hospital (HCH queue) from January 2017 to October 2022. At the same time, we included 487 EC patients who met the inclusion and exclusion criteria in Tongji Hospital (January 2017 to December 2020) as an external validation queue (TJ queue). Inclusive criteria: (1) patients with complete clinical data and ≥18 years old; (2) all patients were initially treated with surgery, and were pathologically diagnosed as EC after surgery. Exclusion criteria: (1) patients with diseases that affect serum sex hormone levels; (2) patients with liver and kidney dysfunction; (3) patients who received chemotherapy, radiotherapy, hormone therapy and molecular targeted therapy before surgery. This study complies with the Declaration of Helsinki, and has been approved by the Ethics Committee of Hubei Cancer Hospital (LLHBCH2022YN-043) and the Ethics Committee of Tongji Hospital, Tongji Medical College, Huazhong University of Science and Technology (TJ-IRB20220556) at the beginning of this study. As this study was a retrospective study, the included patient data was anonymous, so the requirement for informed consent was waived. The flow chart of patient inclusion, model construction and verification were summarized in Figure 1.

### 2.2. Clinical data acquisition and quality control

We obtained the patient’s clinical information by consulting the patient’s electronic medical record, including age, menopausal status, family history of tumor, pathological type, degree of differentiation, lymph node metastasis (LNM), preoperative serum carbohydrate antigen 125, preoperative serum epididymal protein 4, preoperative serum albumin, preoperative D-dimer, preoperative ovarian stimulating hormone, luteinizing hormone, testosterone, progesterone, prolactin and estradiol, and blood routine (neutrophil count, lymphocyte count, monocyte count, etc). For data quality control, we used the maximum likelihood method to perform iterative operations to fill in missing values and estimate parameters, that is, the first step (“prediction step”), given an estimate of an unknown parameter, predicts the missing data in the sufficient statistics; In the second step (“estimation step”), use the sufficient statistics obtained in this step to calculate the correction value of the maximum likelihood estimation of the parameter, and repeat the above 2 steps until the results of the 2 previous calculations reach the specified convergence standard.

### 2.3. Evaluation criteria for ovarian metastasis of EC

The diagnostic criteria for ovarian metastasis of EC were proposed according to the existing literature, namely: (1) ovarian tumors were multinodular, and tumor nodules could be seen in medulla and ovarian cortex under microscope; (2) ovarian tumors with 2 or more of the following conditions: bilateral ovarian invasion, fallopian tube involvement, ovarian diameter > 5 cm, vascular invasion, deep muscle layer infiltration; During operation, cancer focus or abnormal ovarian shape can be seen with naked eyes, which is a dominant metastasis; No abnormality was found with naked eyes during the operation, but the pathological examination after the operation confirmed that ovarian metastasis was a recessive metastasis. The dominant and recessive metastasis were ovarian metastasis.

### 2.4. Construction and verification of machine learning model

We randomly divided the patients from Hubei Cancer Hospital into training set (70%) and internal verification set (30%), and constructed the following 5 machine learning models: random forest model (RFM), gradient elevator model (GEM), support vector machine model, artificial neural network model (ANNM), and decision tree model (DTM).^[12–14]^ At the same time, we used the patient data from Tongji Hospital as the external validation queue to conduct ten-fold cross validation model training and validation on the model training dataset and the external validation dataset respectively; In addition, the predictive efficacy of 5 machine learning models in predicting ovarian metastasis of EC was evaluated by receiver operating characteristic curve and decision curve analysis.

### 2.5. Statistical analysis

All data processing, statistical analysis and mapping are carried out in R 4.0.5 software (download website link: https://cran.r-project.org/). For descriptive analysis, the median (inter-quartile range) and frequency (%) of continuous variables and categorical variables were evaluated, respectively. Pearson correlation coefficient is used to evaluate the correlation between 2 continuous variables. Chi square test was used to compare categorical variables, and Wilcoxon rank sum test or *T* test was used to compare continuous variables. All statistical tests are bidirectional. *P* < .05 was considered statistically significant.

## 3. Results

### 3.1. Analysis of clinicopathological parameters of EC patients with ovarian metastasis

In the data set (i.e., HCH queue) where we built the prediction model, 536 patients were randomly divided into training set (N = 375, 70%) and internal validation set (N = 161, 30%) for internal validation, as shown in Table 1. The incidence of ovarian metastasis of EC in training set and internal verification set was 10.4% and 7.45%, respectively. At the same time, we also included 487 cases (namely, TJ cohort) in the external data set, and the incidence of ovarian metastasis of EC was 8.62%. There was no significant difference between the training set and the internal validation set in terms of age, menopause status, family history of tumor, pathological type, D-D, FSH, T, P, E2, and PRL (*P* > .05). Compared with TJ cohort, the above indicators in HCH cohort showed no statistical difference (*P* > .05), as shown in Table S1, Supplemental Digital Content, http://links.lww.com/MD/K136. However, there were significant differences between the 2 groups in tumor differentiation, LNM, CA125, HE4, Alb and LH (*P* < .05).

**Table 1:** Comparison of clinicopathological parameters between ovarian metastasis and non-metastasis in patients with EC (from HCH queue).

Variables	Training set			*P*-value	Internal validation set			*P*-value
	Overall (N = 375)	No (N = 336)	Yes (N = 39)		Overall (N = 161)	No (N = 149)	Yes (N = 12)	
Age (median [IQR]), year	59.00 [46.00, 68.50]	59.00 [46.00, 68.25]	59.00 [52.00, 68.50]	.62	58.00 [48.00, 70.00]	58.00 [49.00, 71.00]	53.00 [44.50, 63.25]	.371
BMI (median [IQR]), kg/m^2^	25.00 [22.10, 27.55]	25.10 [22.20, 27.63]	24.80 [20.50, 27.10]	.236	24.20 [21.50, 27.10]	24.40 [21.50, 27.10]	23.25 [22.80, 24.13]	.503
Menopause (%)								
Yes	326 (86.9)	293 (87.2)	33 (84.6)	.839	144 (89.4)	135 (90.6)	9 (75.0)	.229
No	49 (13.1)	43 (12.8)	6 (15.4)		17 (10.6)	14 (9.4)	3 (25.0)	
Smoking (%)								
Yes	68 (18.1)	58 (17.3)	10 (25.6)	.286	18 (11.2)	16 (10.7)	2 (16.7)	.88
No	307 (81.9)	278 (82.7)	29 (74.4)		143 (88.8)	133 (89.3)	10 (83.3)	
FHT (%)								
Yes	29 (7.7)	24 (7.1)	5 (12.8)	.347	8 (5.0)	6 (4.0)	2 (16.7)	.212
No	346 (92.3)	312 (92.9)	34 (87.2)		153 (95.0)	143 (96.0)	10 (83.3)	
FIGO (%)								
IA	53 (14.1)	47 (14.0)	6 (15.4)	.014	17 (10.6)	16 (10.7)	1 (8.3)	.93
IB	239 (63.7)	215 (64.0)	24 (61.5)		115 (71.4)	106 (71.1)	9 (75.0)	
II	13 (3.5)	12 (3.6)	1 (2.6)		7 (4.3)	6 (4.0)	1 (8.3)	
IIIA	14 (3.7)	14 (4.2)	0 (0.0)		7 (4.3)	7 (4.7)	0 (0.0)	
IIIB	5 (1.3)	5 (1.5)	0 (0.0)		1 (0.6)	1 (0.7)	0 (0.0)	
IIIC1	24 (6.4)	16 (4.8)	8 (20.5)		5 (3.1)	4 (2.7)	1 (8.3)	
IIIC2	16 (4.3)	16 (4.8)	0 (0.0)		4 (2.5)	4 (2.7)	0 (0.0)	
IVA	5 (1.3)	5 (1.5)	0 (0.0)		3 (1.9)	3 (2.0)	0 (0.0)	
IVB	6 (1.6)	6 (1.8)	0 (0.0)		2 (1.2)	2 (1.3)	0 (0.0)	
Pathological type (%)								
Adenocarcinoma	290 (77.3)	263 (78.3)	27 (69.2)	.282	134 (83.2)	127 (85.2)	7 (58.3)	.046
Non-adenocarcinoma	85 (22.7)	73 (21.7)	12 (30.8)		27 (16.8)	22 (14.8)	5 (41.7)	
DTD (%)								
Moderate-low	75 (20.0)	43 (12.8)	32 (82.1)	<.001	26 (16.1)	16 (10.7)	10 (83.3)	<.001
High	300 (80.0)	293 (87.2)	7 (17.9)		135 (83.9)	133 (89.3)	2 (16.7)	
LNM (%)								
Negative	319 (85.1)	315 (93.8)	4 (10.3)	<.001	140 (87.0)	139 (93.3)	1 (8.3)	<.001
Positive	56 (14.9)	21 (6.2)	35 (89.7)		21 (13.0)	10 (6.7)	11 (91.7)	
CA125 (median [IQR]), U/mL	31.90 [30.15, 33.20]	31.55 [29.98, 32.80]	37.80 [33.70, 40.90]	<.001	31.10 [29.30, 33.10]	30.70 [29.20, 32.60]	42.15 [37.70, 44.42]	<.001
HE4 (median [IQR]), pmol/L	143.80 [122.65, 160.10]	140.85 [119.80, 155.70]	198.60 [173.30, 231.70]	<.001	136.80 [119.20, 156.60]	134.60 [118.80, 150.50]	201.30 [188.55, 222.25]	<.001
Alb (median [IQR]), g/L	39.40 [37.45, 41.60]	39.90 [37.90, 41.70]	33.00 [28.30, 35.95]	<.001	39.40 [37.70, 41.70]	39.80 [38.00, 41.80]	32.00 [29.28, 36.25]	<.001
DD (median [IQR]), mg/L	0.72 [0.63, 0.81]	0.72 [0.63, 0.80]	0.74 [0.64, 0.84]	.119	0.72 [0.64, 0.80]	0.71 [0.64, 0.79]	0.82 [0.74, 0.87]	.01
FSH (median [IQR]), mIU/L	9.72 [8.93, 10.45]	9.68 [8.93, 10.36]	10.62 [9.07, 11.30]	.007	9.78 [8.75, 10.54]	9.78 [8.70, 10.48]	10.82 [9.27, 11.25]	.026
LH (median [IQR]), IU/L	9.00 [8.44, 9.66]	8.90 [8.38, 9.44]	11.83 [11.13, 12.61]	<.001	8.97 [8.54, 9.61]	8.93 [8.51, 9.47]	12.07 [11.18, 12.75]	<.001
T (median [IQR]), ng/dL	2.70 [2.52, 2.89]	2.71 [2.52, 2.89]	2.60 [2.41, 2.91]	.119	2.71 [2.56, 2.91]	2.72 [2.56, 2.91]	2.59 [2.53, 2.69]	.066
*P* (median [IQR]), ng/L	1.45 [1.32, 1.58]	1.46 [1.33, 1.58]	1.38 [1.24, 1.60]	.174	1.49 [1.30, 1.62]	1.49 [1.30, 1.63]	1.39 [1.35, 1.55]	.277
E2 (median [IQR]), pmol/L	53.50 [48.70, 58.90]	53.65 [48.88, 59.00]	50.40 [44.35, 57.40]	.01	52.40 [47.60, 56.80]	52.50 [47.60, 56.80]	51.75 [48.95, 56.72]	.745
PRL (median [IQR]), ng/mL	19.50 [17.80, 21.10]	19.40 [17.70, 21.02]	20.30 [18.80, 21.35]	.021	19.60 [18.10, 20.80]	19.50 [18.00, 20.80]	19.75 [19.17, 21.28]	.329
NLR (median [IQR])	2.72 [2.26, 3.16]	2.74 [2.30, 3.20]	2.40 [2.05, 3.05]	.045	2.64 [2.17, 3.21]	2.62 [2.17, 3.21]	2.74 [2.33, 2.94]	.954
NAR (median [IQR])	0.12 [0.11, 0.13]	0.12 [0.11, 0.13]	0.11 [0.11, 0.12]	.802	0.12 [0.11, 0.12]	0.12 [0.11, 0.12]	0.11 [0.11, 0.13]	.759
PLR (median [IQR])	120.30 [105.80, 132.40]	120.40 [106.20, 132.30]	116.00 [99.05, 133.25]	.332	118.30 [105.50, 131.70]	119.00 [106.70, 132.00]	110.95 [95.30, 118.55]	.023
LMR (median [IQR])	4.50 [4.00, 5.10]	4.50 [4.00, 5.20]	4.10 [3.80, 4.80]	.008	4.50 [3.90, 5.10]	4.50 [3.90, 5.10]	4.60 [4.30, 4.75]	.969

BMI = body mass index; DTD = degree of tumor differentiation; FHT = family history of tumor; FIGO = International Federation of Gynecology and Obstetrics; IQR = inter-quartile range; LMR = lymphocyte-to-monocyte ratio; LNM = lymph node metastasis; NAR = neutrophil-to-albumin ratio; NLR = neutrophil-to-lymphocyte ratio; PLR = platelet-to-lymphocyte ratio.

### 3.2. Selection of predictive variables for ovarian metastasis of EC

**Figure 2.**
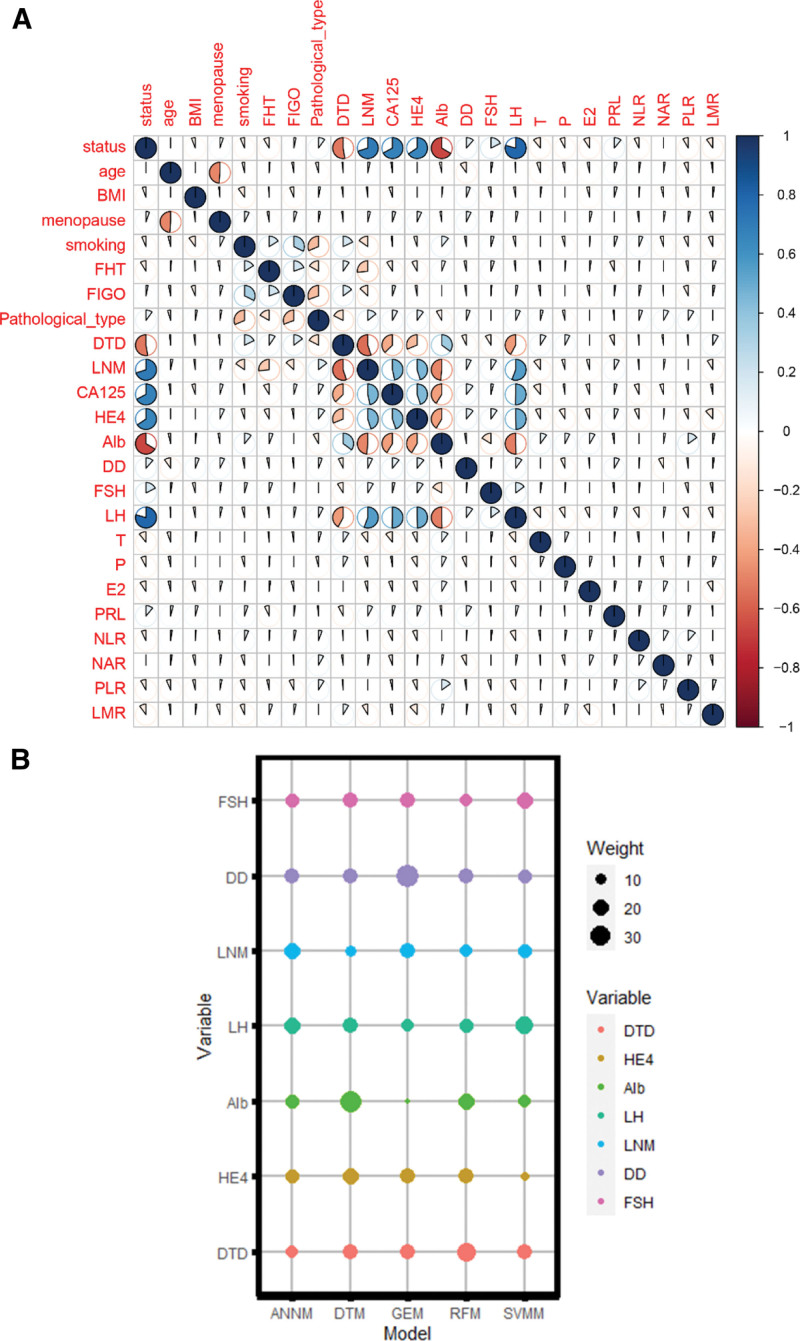
Screening of candidate variables for prediction model of ovarian metastasis of EC. (A) Correlation analysis between ovarian metastasis and candidate variables; (B) weight assignment of candidate variables in 5 machine learning algorithms.

For screening of candidate predictors of ovarian metastasis of EC, we adopted a two-step analysis, namely: Pearson product-moment correlation coefficient, as shown in Figure 2A. We analyzed the correlation between all the variables that can be included and the outcome variables (that is, whether the ovary is metastatic or not as a “second category”). The results showed that the degree of tumor differentiation, LNM, CA125, HE4, Alb, LH, and ovarian metastasis had a strong direct correlation (that is, *r* coefficient). In view of this, we immediately carried out “weight diversion” analysis on candidate variables with significant positive or negative correlation in the prediction models of 5 machine learning algorithms (Fig. 2B). The results showed that the above candidate variables accounted for a certain proportion in various machine learning prediction models, indicating the degree of tumor differentiation, LNM, CA125, HE4, Alb LH can be used as a potential predictor of ovarian metastasis prediction model in EC patients.

### 3.3. Construction of predictive model for ovarian metastasis of EC based on machine learning algorithm

**Figure 3.**
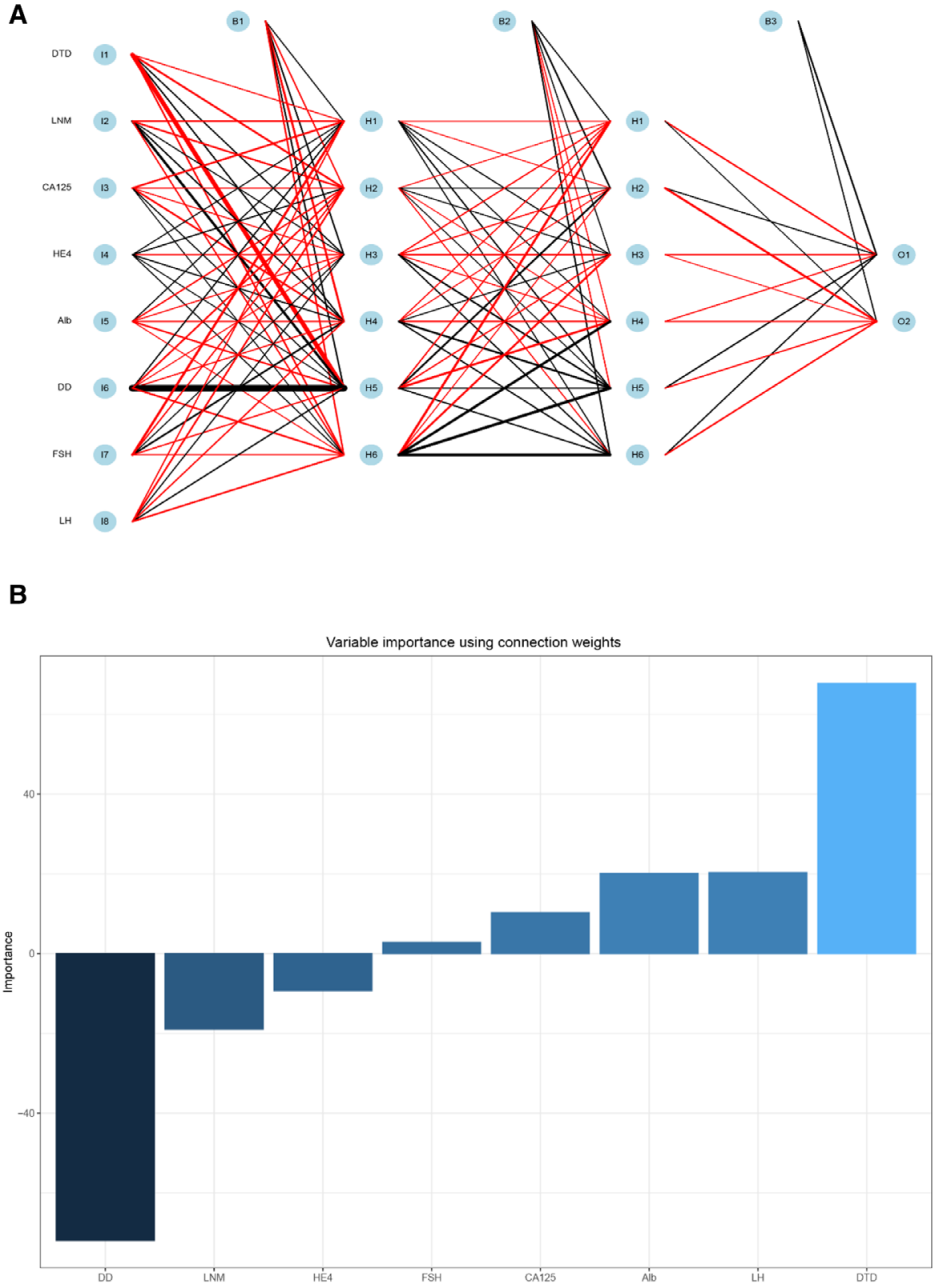
Prediction model of ovarian metastasis in patients with EC based on ANN algorithm. (A) Construction of ANNM; (B) proportion of weight coefficient distribution of ANNM candidate variables.

**Figure 4.**
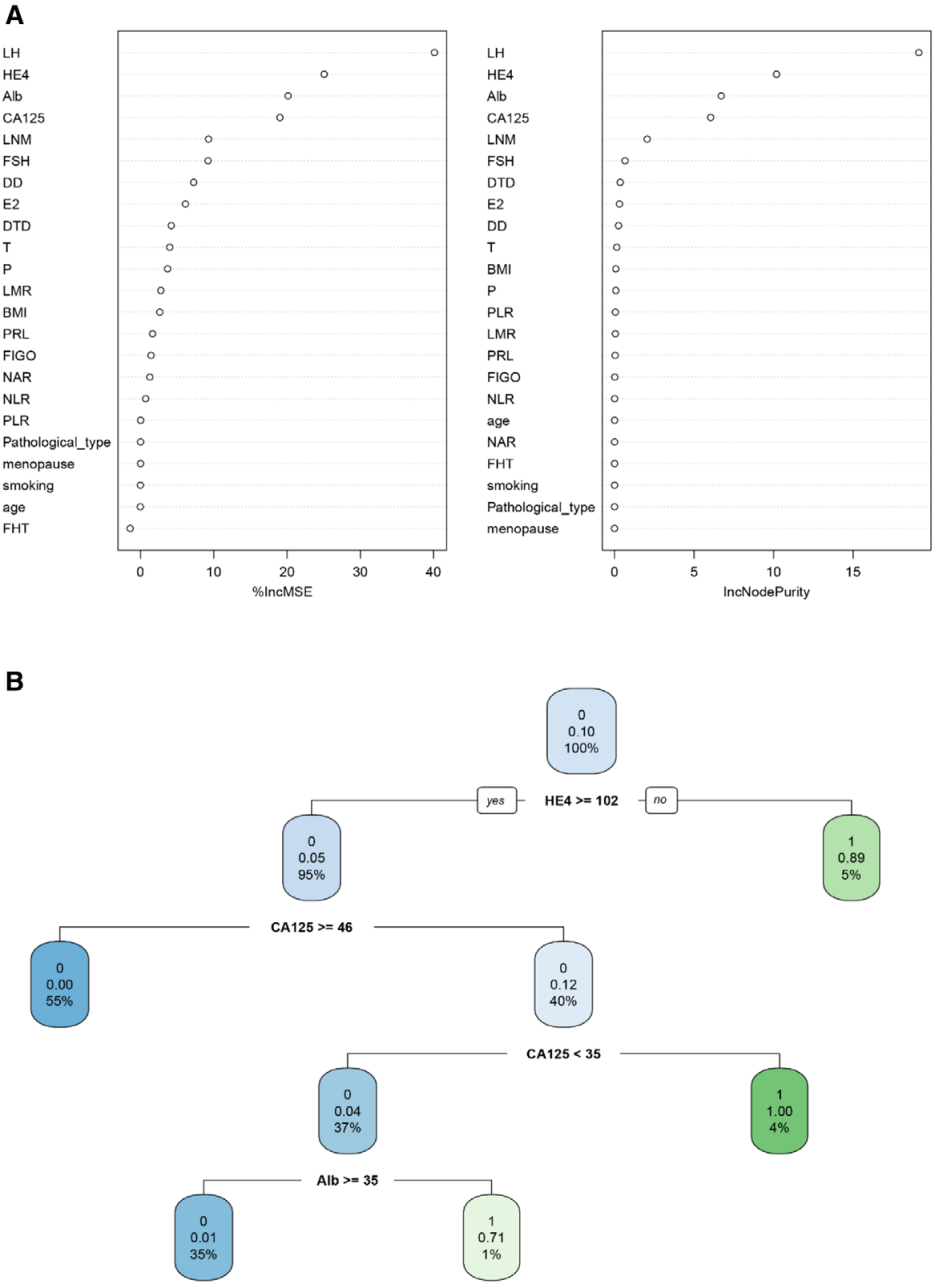
Prediction model of ovarian metastasis in patients with EC based on “Decision Tree” algorithm. (A) RFM; (B) DTMM.

For different machine learning algorithm rules, we respectively build prediction models for RFM, ANNM, DTM, GEM, and SVM. For example, in the ANNM model, based on the algorithm called “Multilayer Feed-Forward Neural Network.”^[15]^ We included the degree of tumor differentiation, LNM, CA125, HE4, Alb, LH (that is, the input layer), carried out “hidden layer” iterative analysis, and finally obtained the hierarchical effect of ovarian metastasis risk (Fig. 3); In the RFM model, based on its “bagging” algorithm, we take the included candidate variables as “branching” nodes, and then conduct iterative analysis (Fig. 4A and Table S2, Supplemental Digital Content, http://links.lww.com/MD/K137), that is, the included variables are branched according to “Ensemble learning,” and finally get the prediction results.^[16]^ On this basis, DTM model will be regarded as the same kind of algorithm of RFM, while SVM follows the principle of “approximating discrete function value,” as shown in Figure 4B. Similarly, SVM and GEM also follow their own algorithm classification principles. In a word, based on different machine learning algorithms, we have built a practical prediction model that can be used to predict ovarian metastasis of EC patients.

### 3.4. Effectiveness evaluation of prediction models constructed by different algorithms

**Figure 5.**
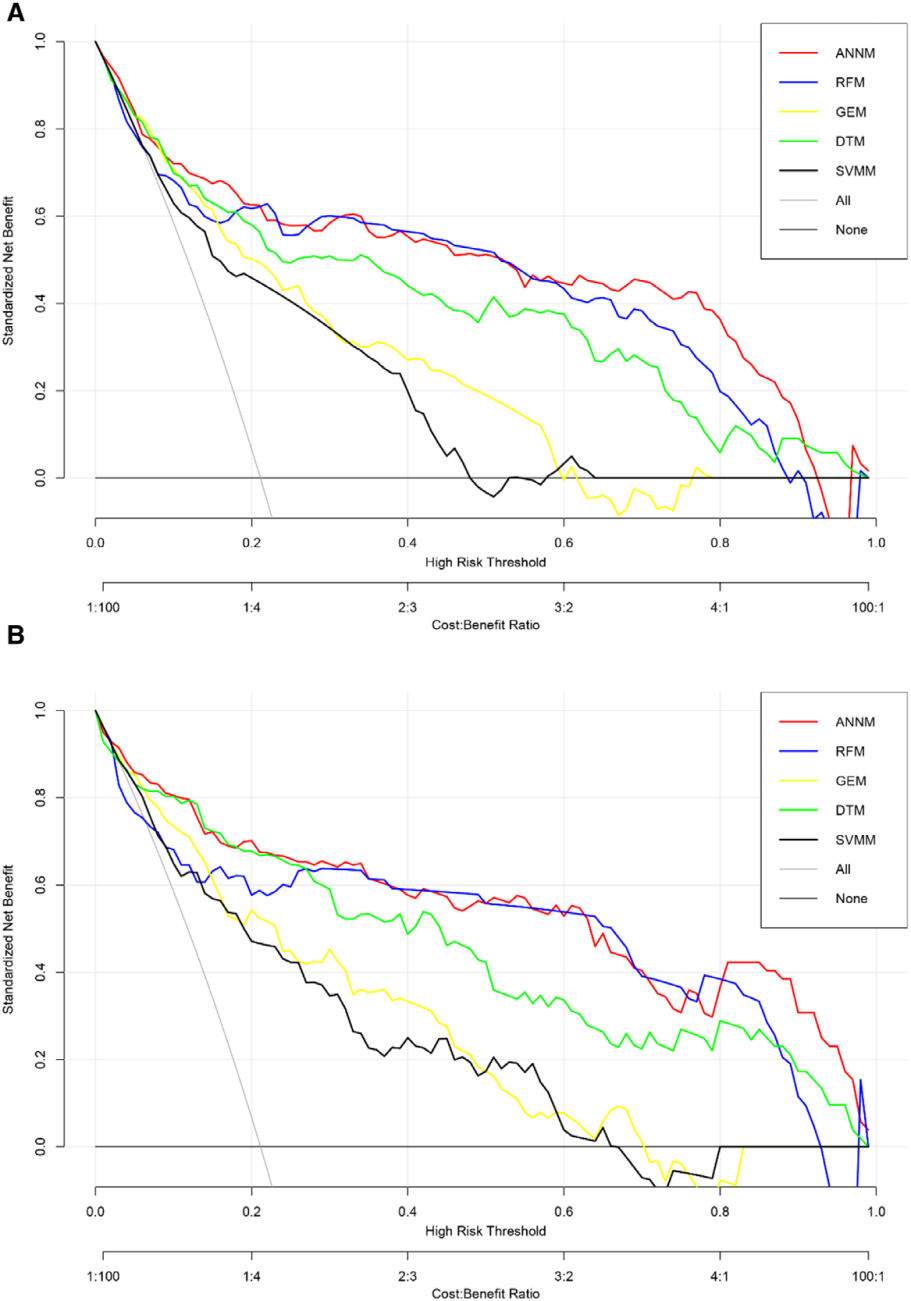
Effectiveness evaluation of prediction model constructed by machine learning algorithm. (A) Training set; (B) internal validation set.

To further evaluate the robustness and accuracy of various prediction models in predicting ovarian metastasis of EC patients, we used decision curve analysis to evaluate the effectiveness of 5 machine learning prediction models. That is, the function calculates the decision curve, which is the estimation of the standardized net income according to the probability threshold, and is used to classify the observation results as “high risk.”^[17]^ As shown in Figure 5, the robustness of ANNM is significantly better than the other 4 prediction models in both training set and validation set queues, which shows that ANNM has certain advantages in predicting ovarian metastasis of endometrial cancer patients. At the same time, we also used the traditional receiver operating characteristic to evaluate the accuracy. The results showed that the diagnostic effectiveness of ANNM in training set and internal verification set was (area under curve [AUC]: 0.899, 95% confidence interval [CI]: 0.844–0.954) and (AUC: 0.892, 95% CI: 0.837–0.947), respectively, followed by the RFM model (training set: [AUC: 0.826, 95% CI: 0.771–0.881]; verification set: [AUC: 0.831, 95% CI: 0.776–0.886]), as shown in Table 2. In a word, the 5 machine learning algorithm ovarian metastasis prediction models have satisfactory prediction efficiency, and can provide risk stratification guidance for EC patients with ovarian metastasis.

**Table 2:** The receiver operating characteristic curve analyses for ovarian metastasis in each machine learning-based prediction model.

Model	Training set			Internal validation set			External validation set		
	AUC mean	AUC 95%CI	Variables*	AUC mean	AUC 95%CI	Variables*	AUC mean	AUC 95%CI	Variables*
ANNM	0.899	0.844–0.954	7	0.892	0.837–0.947	7	0.887	0.832–0.942	7
RFM	0.826	0.771–0.881	7	0.831	0.776–0.886	7	0.828	0.773–0.883	7
DTM	0.796	0.741–0.851	5	0.793	0.738–0.848	5	0.789	0.734–0.844	5
GEM	0.764	0.709–0.819	8	0.771	0.716–0.826	8	0.772	0.717–0.827	8
SVMM	0.729	0.674–0.784	7	0.732	0.677–0.787	7	0.727	0.672–0.782	7

* Variables included in the prediction model.

### 3.5. External queue verification of prediction models constructed by different algorithms

**Figure 6.**
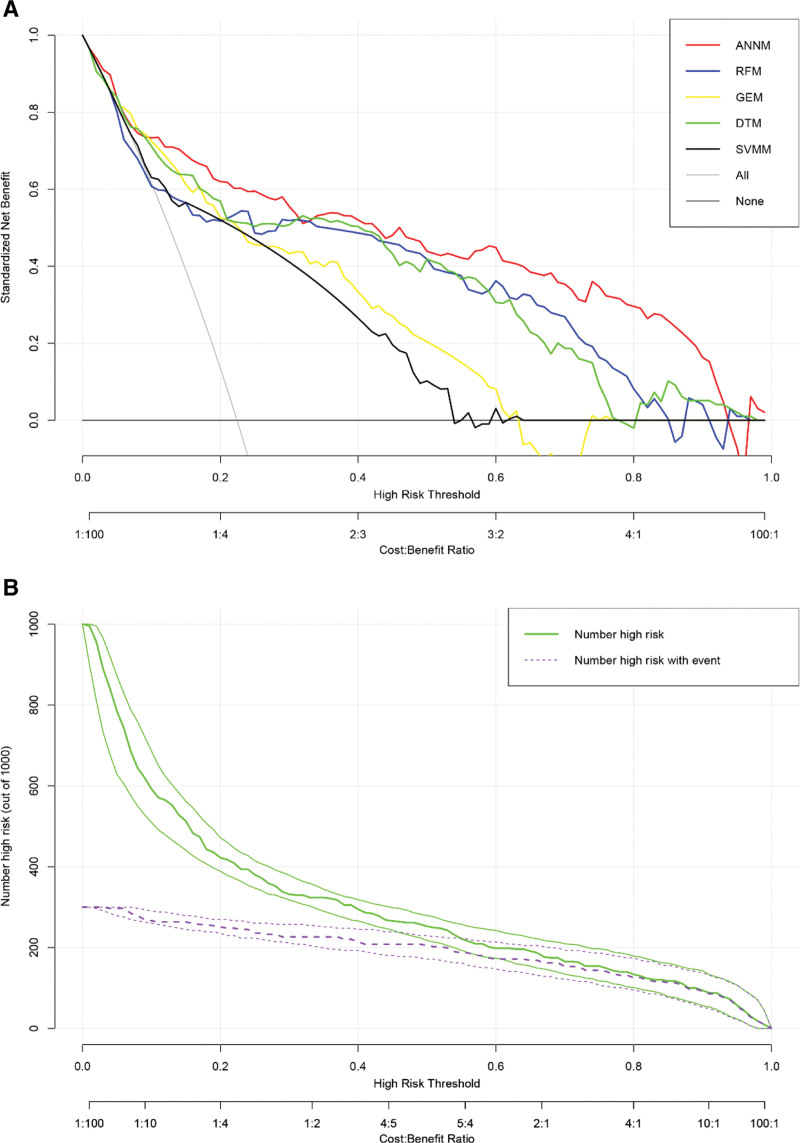
External queue verification of prediction model constructed by machine learning algorithm. (A) Verification of robustness and prediction efficiency of prediction model (from TJ queue); (B) identification efficiency of ANNM in differentiating ovarian metastasis.

In order to continue tracking and evaluating the prediction effectiveness of machine learning models in external queues, we immediately took TJ queue as the external verification of this study. As shown in Figure 6A, the prediction models of 5 machine learning algorithms still have consistent robustness in external queues. Similarly, we also used the optimal prediction model (i.e., ANNM) to predict the hierarchical risk of ovarian metastasis in the TJ cohort, as shown in Figure 6B. Even in the external cohort, ANNM can also identify whether patients have ovarian metastasis risk, which confirms that the ovarian metastasis prediction model of EC has reliable scalability.

## 4. Discussion

EC is characterized by high invasion, metastasis and poor prognosis, among which ovarian metastasis is relatively common.^[18]^ The previous epidemiological data showed that the ovarian metastasis rate of EC was between 9.7% and 13.56%.^[6,19]^ This study was consistent with the previous report, that is, the planting and spreading rate of cancer on the ovary was between 8.53% and 11.8%. Therefore, it is of great significance to study the risk factors of ovarian metastasis of EC for clinical measures to reduce the probability of ovarian metastasis of EC patients. In this study, the prediction model of ovarian metastasis based on machine learning algorithm was constructed according to the risk factor indicators of EC patients with ovarian metastasis. It was found that the prediction model (especially ANNM) has high sensitivity and specificity, which may be a combination of multiple indicators to achieve information complementation, enhancing the sensitivity and specificity of the model, thus effectively improving the prediction efficiency of EC patients with ovarian metastasis, so it can be better applied to guide clinical practice.

Previous studies have shown that ovarian metastasis of EC is usually caused by the interaction of multiple factors.^[19–21]^ The results of this study showed that tumor differentiation, LNM, CA125, Alb, HE4, and LH were independent risk factors for ovarian metastasis of EC (*P* < .05). The reason for this may be that malignant tumor cells have more or less the characteristics of differentiation to normal cells.^[22,23]^ The closer the tumor cells are to normal cells, the higher the degree of differentiation, and the lower the degree of malignancy; On the contrary, the greater the difference with normal cells, the lower the degree of differentiation, the more active the proliferation and growth of cancer cells, and the higher the degree of malignancy. Therefore, the higher the degree of differentiation, the worse its invasion. The lower the degree of differentiation, the stronger its invasion ability, and the higher the risk of ovarian metastasis. Under normal circumstances, the LNM rate of EC patients with good differentiation and muscle layer infiltration depth < 1/2 is relatively low.^[24]^ However, if EC patients have LNM and involvement, their risk of ovarian metastasis will be greatly increased, because cancer cells can invade the ovary through the cancer focus at the bottom of the uterus along the lymphatic network above the broad ligament, and through the pelvic infundibulum ligament, anastomose with the lower ovarian collecting lymphatic vessels, retrogradely invade the ovary and ectopic planting.^[25,26]^

CA125 is a high molecular weight transmembrane glycoprotein encoded by MUC16 gene, which is rarely secreted or not secreted by human body under normal conditions.^[27,28]^ The level of CA125 is related to many factors, such as the inflammatory reaction of the body, the progress of malignant tumor, the amount of ascites, etc. When endometrial malignant lesions appear, the serum level can be abnormally elevated. When cancer cells metastasize to other tissues or organs and continue to proliferate, the secretion of CA125 antigen will increase.^[29]^ Some studies have shown that the positive rate of CA125 in serum of patients with EC who have cancer metastasis in their appendages or positive peritoneal cytology is significantly increased.^[29–31]^ This may be because the endometrial barrier is seriously damaged by cancer cells, which provides convenient conditions for tumor cells to transfer to Miller tubes or mesothelial tissues such as ovaries and fallopian tubes, thus increasing the expression level of CA125. In addition, HE4 is a secreted glycoprotein encoded by whey acidic protein gene.^[32]^ Under physiological conditions, it is mainly expressed in the respiratory system and reproductive system, and the expression level is related to age, smoking, and menstrual cycle.^[32,33]^ Under the pathological conditions of malignant tumors, HE4 can induce EC cell proliferation and promote tumor cell metastasis and invasion by activating related carriers and signal pathways. In addition, the levels of CA125 and HE4 will increase with the decrease of tumor differentiation, and the serum levels of CA125 and HE4 in patients with LNM are higher than those without LNM. To sum up, this study suggests that serum CA125 and HE4 levels can reflect whether lymph nodes have micrometastasis to some extent.

Alb has many physiological functions such as maintaining colloid osmotic pressure in blood vessels, anti-inflammation, anti-oxidation, and scavenging oxygen free radicals. The development process of malignant tumor is closely related to the metabolic disorder of human body.^[34,35]^ With the development of the disease, protein catabolism increases and anabolism decreases, leading to negative nitrogen balance.^[35]^ In addition, during the development of cancer, the permeability of microvessels will increase, and the penetration of Alb will increase, which can further reduce the content of Alb.^[36]^ The role of LH is to promote ovarian ovulation and luteinization, and promote luteal secretion of estrogen and progesterone.^[37]^ Estrogen and progesterone can regulate the cytokines and growth factors in the endometrium and exert influence on the endometrium, stroma and perivascular.^[38,39]^ In vitro studies have confirmed that progesterone can inhibit the release of cytokines from mouse and human EC cells, estrogen can induce the activation of nuclear factors, and increase the proliferation and migration capacity of Ishikawa cells.^[40]^ In addition, androgen receptors can also express in endometrium at different stages of the menstrual cycle, postmenopausal atrophic endometrium, precancerous lesions, and develop into EC, so as to have an anti-proliferative effect on endometrial cells.^[41]^ However, under the effect of cancer cells, the receptivity of endometrium and pelvic microenvironment will change significantly, and can show periodic changes under the influence of estrogen and progesterone, the cancer cell differentiation factor will release and retrograde into the fallopian tube, ovary and other parts to form ectopic planting.

In this study, 5 types of ovarian metastasis prediction models based on machine learning algorithm and the above available clinical indicators can be used for layered diagnosis and treatment of EC patients. In recent years, machine learning algorithm has been widely used in the medical field because of its superior characteristics of “iteration and “progression.”^[42,43]^ Interestingly, this study also found that the optimal prediction model ANNM can greatly improve the prediction efficiency of ovarian metastasis in EC patients. This may be due to the fact that after correcting the weights of various neurons in the network, the network will be output in the forward propagation mode again, resulting in the error between the actual output value and the expected value, which can lead to a new round of weight correction.^[44,45]^ Finally, the process of forward propagation and back propagation repeats until the network converges, and the interconnection weight and threshold value after the network converges are obtained, making the output result more robust.

Although the clinical significance of machine learning algorithm in predicting ovarian metastasis of EC is promising, some limitations should also be recognized. First, all the samples in this study were retrospective, and the validation of future prediction models should be conducted in a prospective multicenter cohort. Second, this study was slightly monotonous in screening predictors based on clinical pathological parameters and serological indicators. In the future, we still need to use multi group technology (such as blood, urine proteomics, transcriptomics, etc) to find a better prediction marker to build a more robust prediction model. Third, the sample size based on this study was still small, and there was an inevitable risk of data imputation loss and selection bias. In the future, large sample data will still be required for multi-dimensional verification and optimization.

## 5. Conclusion

In conclusion, based on the multi-dimensional machine learning algorithm, we developed a stable and powerful prediction model for evaluating ovarian metastasis of EC. In particular, ANNM is undeniably a powerful forecasting tool that can help determine optimal clinical management strategies.

## Acknowledgments

The authors thank all study participants for consenting to the use of their medical records.

## Author contributions

**Investigation:** Qin Zhao, Wang Tiejun.

**Methodology:** Qin Zhao, Yinuo Li, Wang Tiejun.

**Resources:** Qin Zhao, Wang Tiejun.

**Software:** Yinuo Li.

**Supervision:** Qin Zhao, Yinuo Li, Wang Tiejun.

**Visualization:** Wang Tiejun.

**Writing – original draft:** Yinuo Li.

**Writing – review & editing:** Wang Tiejun.

## Supplementary Material
