# Supplementary material for: Development and validation of prediction model for early warning of ovarian metastasis risk of endometrial carcinoma
Source: Medicine (Baltimore). 2023 Oct 13;102(41):e35439. doi: 10.1097/MD.0000000000035439 (PMC10578755; doi:10.1097/MD.0000000000035439)
Supplement: Supplementary file 1 [file medi-102-e35439-s001.docx]

Supplementary Table1. Comparison of clinicopathological parameters between ovarian metastatic and non metastatic patients with endometrial carcinoma (from TJ cohort)

| Variables | Overall(N=487) | Yes(N=42) | No(N=445) | P-value |
| --- | --- | --- | --- | --- |
| Age (median [IQR]),year | 55.00 [45.00, 65.00] | 49.50 [40.25, 59.75] | 55.00 [45.00, 65.00] | 0.014 |
| BMI (median [IQR]),kg/m^2^ | 25.70 [23.35, 28.30] | 25.25 [23.85, 27.98] | 25.70 [23.30, 28.40] | 0.756 |
| Menopause (%) |  |  |  |  |
| yes | 362 (74.3) | 26 (61.9) | 336 (75.5) | 0.081 |
| no | 125 (25.7) | 16 (38.1) | 109 (24.5) |  |
| Smoking (%) |  |  |  |  |
| yes | 65 (13.3) | 8 (19.0) | 57 (12.8) | 0.369 |
| no | 422 (86.7) | 34 (81.0) | 388 (87.2) |  |
| FHT (%) |  |  |  |  |
| yes | 19 (3.9) | 5 (11.9) | 14 (3.1) | 0.017 |
| no | 468 (96.1) | 37 (88.1) | 431 (96.9) |  |
| FIGO (%) |  |  |  |  |
| IA | 59 (12.1) | 4 (9.5) | 55 (12.4) | <0.001 |
| IB | 340 (69.8) | 27 (64.3) | 313 (70.3) |  |
| II | 11 (2.3) | 0 (0.0) | 11 (2.5) |  |
| IIIA | 7 (1.4) | 0 (0.0) | 7 (1.6) |  |
| IIIB | 10 (2.1) | 0 (0.0) | 10 (2.2) |  |
| IIIC1 | 15 (3.1) | 9 (21.4) | 6 (1.3) |  |
| IIIC2 | 11 (2.3) | 2 (4.8) | 9 (2.0) |  |
| IVA | 9 (1.8) | 0 (0.0) | 9 (2.0) |  |
| IVB | 15 (3.1) | 0 (0.0) | 15 (3.4) |  |
| IVC | 10 (2.1) | 0 (0.0) | 10 (2.2) |  |
| Pathological type (%) |  |  |  |  |
| adenocarcinoma | 396 (81.3) | 31 (73.8) | 365 (82.0) | 0.272 |
| non-adenocarcinoma | 91 (18.7) | 11 (26.2) | 80 (18.0) |  |
| DTD (%) |  |  |  |  |
| moderate-low | 82 (16.8) | 37 (88.1) | 45 (10.1) | <0.001 |
| high | 405 (83.2) | 5 (11.9) | 400 (89.9) |  |
| LNM (%) |  |  |  |  |
| negative | 436 (89.5) | 3 (7.1) | 433 (97.3) | <0.001 |
| Positive | 51 (10.5) | 39 (92.9) | 12 (2.7) |  |
| CA125 (median [IQR]), U/mL | 31.80 [30.00, 33.50] | 38.15 [34.80, 42.30] | 31.60 [29.70, 33.00] | <0.001 |
| HE4 (median [IQR]), pmol/L | 145.90 [129.00, 165.40] | 201.25 [182.10, 225.75] | 143.00 [128.10, 161.10] | <0.001 |
| Alb (median [IQR]), g/L | 39.90 [37.60, 41.80] | 33.30 [28.73, 36.27] | 40.10 [38.10, 41.90] | <0.001 |
| DD (median [IQR]), mg/L | 0.74 [0.64, 0.83] | 0.74 [0.64, 0.82] | 0.74 [0.64, 0.83] | 0.773 |
| FSH (median [IQR]), mIU/L | 9.77 [8.72, 10.44] | 10.35 [8.65, 11.06] | 9.76 [8.72, 10.36] | 0.028 |
| LH (median [IQR]), IU/L | 9.06 [8.55, 9.72] | 11.28 [10.75, 12.16] | 8.98 [8.53, 9.49] | <0.001 |
| T (median [IQR]), ng/dL | 2.71 [2.54, 2.89] | 2.62 [2.41, 2.89] | 2.72 [2.54, 2.89] | 0.106 |
| P (median [IQR]), ng/L | 1.40 [1.29, 1.57] | 1.40 [1.28, 1.60] | 1.41 [1.29, 1.57] | 0.715 |
| E2 (median [IQR]), pmol/L | 52.00 [48.10, 58.40] | 52.85 [47.15, 57.75] | 52.00 [48.30, 58.40] | 0.293 |
| PRL (median [IQR]), ng/mL | 19.60 [18.20, 21.30] | 19.90 [18.70, 21.37] | 19.50 [18.20, 21.20] | 0.188 |
| NLR (median [IQR]) | 2.71 [2.26, 3.16] | 2.40 [2.12, 3.08] | 2.74 [2.28, 3.17] | 0.202 |
| NAR (median [IQR]) | 0.12 [0.11, 0.12] | 0.12 [0.11, 0.13] | 0.12 [0.11, 0.12] | 0.664 |
| PLR (median [IQR]) | 116.70 [106.50, 130.30] | 109.50 [97.62, 126.80] | 116.70 [107.40, 130.30] | 0.018 |
| LMR (median [IQR]) | 4.50 [4.00, 5.20] | 4.60 [4.00, 4.90] | 4.50 [4.00, 5.20] | 0.137 |

Abbreviations: IQR. inter-quartile range;BMI. Body mass index;FHT.Family history of tumor;FIGO. International Federation of Gynecology and Obstetrics;DTD. Degree of tumor differentiation; LNM. Lymph node metastasis;NLR. neutrophil-to-lymphocyte ratio;NAR. neutrophil-to-albumin ratio;PLR. platelet-to-lymphocyte ratio;LMR. lymphocyte-to-monocyte ratio.
