# Supplementary material for: Development and validation of prediction model for early warning of ovarian metastasis risk of endometrial carcinoma
Source: Medicine (Baltimore). 2023 Oct 13;102(41):e35439. doi: 10.1097/MD.0000000000035439 (PMC10578755; doi:10.1097/MD.0000000000035439)
Supplement: Supplementary file 2 [file medi-102-e35439-s002.docx]

Supplementary Table2. Weight distribution coefficient of candidate variables in RFM

| Variables | %IncMSE | IncNodePurity |
| --- | --- | --- |
| Age | -0.0323 | 0.006272 |
| BMI | 2.629737 | 0.079882 |
| Menopause | 4.10E-36 | 5.82E-17 |
| Smoking | -0.01612 | 0.003643 |
| FHT | -1.41541 | 0.004336 |
| FIGO | 1.430864 | 0.016226 |
| Pathological_type | 4.16E-36 | 0.001823 |
| DTD | 4.20069 | 0.356067 |
| LNM | 9.283893 | 2.05482 |
| CA125 | 19.03224 | 6.045722 |
| HE4 | 25.08052 | 10.18247 |
| Alb | 20.14703 | 6.70872 |
| DD | 7.256227 | 0.252005 |
| FSH | 9.22355 | 0.662149 |
| LH | 40.13434 | 19.1299 |
| T | 3.986354 | 0.141224 |
| P | 3.693259 | 0.079503 |
| E2 | 6.130394 | 0.311134 |
| PRL | 1.639885 | 0.041844 |
| NLR | 0.705944 | 0.006546 |
| NAR | 1.27209 | 0.005024 |
| PLR | 0.013196 | 0.057699 |
| LMR | 2.774878 | 0.052449 |

Abbreviations: BMI. Body mass index;FHT.Family history of tumor;FIGO. International Federation of Gynecology and Obstetrics;DTD. Degree of tumor differentiation; LNM. Lymph node metastasis;NLR. neutrophil-to-lymphocyte ratio;NAR. neutrophil-to-albumin ratio;PLR. platelet-to-lymphocyte ratio;LMR. lymphocyte-to-monocyte ratio.
